# Venous Thromboembolism in Patients with Inflammatory Bowel Disease

**DOI:** 10.3390/jcm13010251

**Published:** 2023-12-31

**Authors:** Galvin Dhaliwal, Michael V. Patrone, Stephen J. Bickston

**Affiliations:** Division of Gastroenterology, Hepatology and Nutrition, Virginia Commonwealth University, Richmond, VA 23219, USA; michael.patrone@vcuhealth.org (M.V.P.); stephen.bickston@vcuhealth.org (S.J.B.)

**Keywords:** venous thromboembolism, inflammatory bowel disease, VTE, IBD, prophylaxis, Crohn’s disease, ulcerative colitis, surgery, hospitalization

## Abstract

Patients diagnosed with inflammatory bowel disease (IBD), which encompasses Crohn’s disease and ulcerative colitis, experience chronic inflammation of the gastrointestinal tract. Those with IBD face a higher risk of developing venous thromboembolism (VTE) compared to individuals without IBD. This escalated risk is associated with various factors, some modifiable and others non-modifiable, with disease activity being the primary concern. Interestingly, Janus Kinase inhibitors approved for the treatment of IBD may be associated with an increased risk of VTE but only in patients that have other underlying risk factors leading to an overall increased VTE risk. Several recognized medical societies have recommended the use of VTE prophylaxis for hospitalized individuals with IBD. The association between VTE and IBD and the need for pharmacologic prophylaxis remains under-recognized. Increased awareness of this complication can hopefully protect patients from a potentially deadly complication.

## 1. Introduction

Inflammatory bowel disease (IBD), which includes Crohn’s disease and ulcerative colitis, is a chronic inflammatory condition of the gastrointestinal tract. The incidence of IBD is increasing, with a prevalence exceeding 1.3% in the United States [1] and over 0.3% globally [2]. While Crohn’s disease can affect any part of the gastrointestinal tract, ulcerative colitis primarily targets the colon and rectum [3]. Patients with IBD experience various intestinal symptoms, including abdominal pain, loose stools, bleeding per rectum, fecal urgency, nausea, and loss of appetite. Constitutional symptoms may include fever, fatigue, and weight loss. Several individuals develop extra-intestinal manifestations, including involvement of the hepatobiliary system, skin, joints, and eyes, that may progress independently of bowel disease activity. Typically, these symptoms tend to manifest as a relapsing-remitting or chronically progressive pattern [4,5,6].

**Figure 1:**
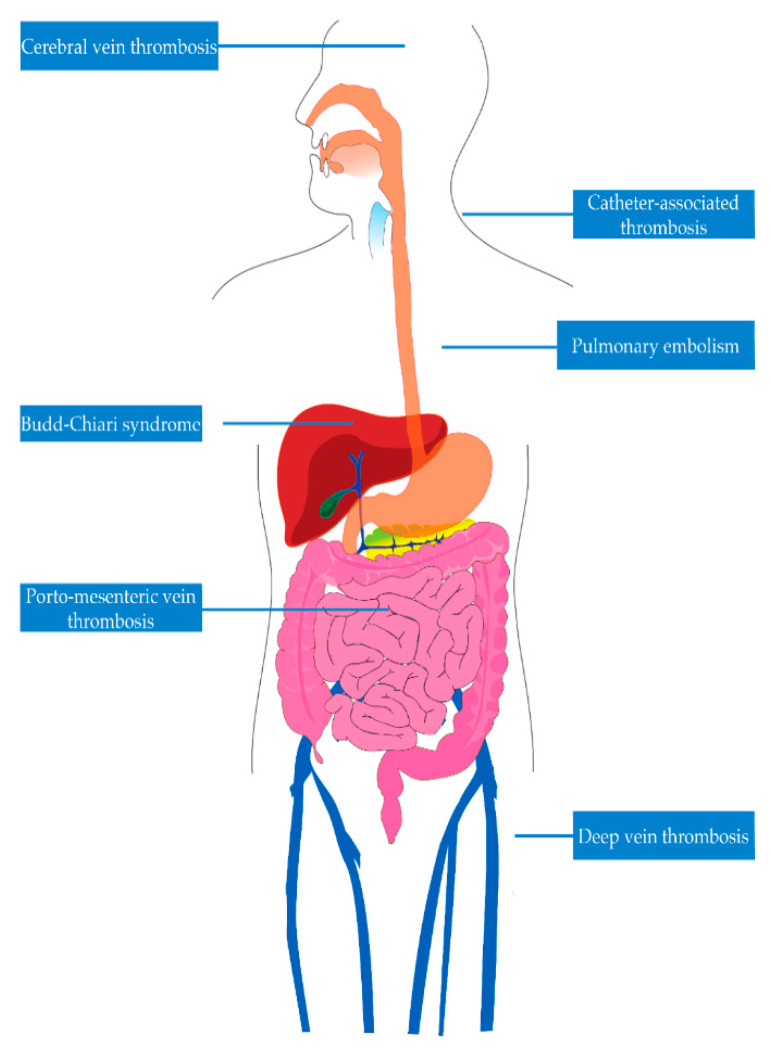
Venous sites of thrombosis in inflammatory bowel disease.

Venous thromboembolism (VTE) is one of the deadliest complications in patients with IBD. VTE is a medical condition characterized by inappropriate formation of blood clots. It encompasses deep vein thrombosis (DVT) and pulmonary embolism (PE). While DVT usually occurs in the deep veins of the lower extremities, it may also occur in deep veins of the upper extremities, mesenteric veins, portal vein, hepatic veins (Budd-Chiari syndrome), and cerebral veins, as seen in Figure 1 [7,8,9,10,11,12,13,14,15]. In the United States, each year, more than half a million hospitalizations have been linked to VTE [16]. Following an initial VTE episode, potential long-term complications encompass post-phlebitic syndrome, pulmonary hypertension, and the likelihood of the condition recurring [17,18,19]. Quality of life can be negatively impacted for up to four months following a DVT. Those with post-thrombotic syndrome endure a further decline in their quality of life during this time, showing changes similar to those found in individuals with chronic heart, lung, or arthritic conditions [20]. Some groups of VTE patients need prolonged anticoagulation to prevent more clots, affecting their quality of life and heightening their risk of bleeding episodes. The total healthcare expenses associated with VTE amount to approximately USD 5–10 billion annually in the United States [3]. As several known risk factors like aging, surgical procedures, immobility, and obesity become more prevalent, VTE emerges as a significant and growing public health concern [21,22,23]. To assess the public’s knowledge regarding DVT, encompassing its symptoms and risk factors, the CDC included DVT-related questions in the 2007 HealthStyles survey [21]. Remarkably, only 38% of respondents accurately identified DVT as a blood clot within a vein. Most participants were unable to identify common risk factors for DVT (SEC, unpublished observations, 2009).

Considering the substantial healthcare and financial burden, we must persist in directing our healthcare endeavors toward the prevention of VTE [3]. Adopting a comprehensive public health approach and promoting awareness about the burden of VTE holds substantial promise in preventing and decreasing morbidity and mortality associated with VTE [21].

This review article intends to outline the current understanding of venous thrombosis in IBD, focusing on its epidemiology, pathophysiology, risk factors, prevention, and treatment.

## 2. Epidemiology

Prior clinical studies have yielded inconsistent findings regarding the prevalence of thromboembolism in IBD, with reported rates ranging from 1.2% to 6.7%, although some postmortem studies have shown figures closer to 40% [24]. Patients diagnosed with IBD have a notably higher susceptibility to VTE compared to the general population [25,26]. Extensive cohort studies and meta-analyses have revealed that individuals with IBD face a 2–3 times increased risk of experiencing deep vein thrombosis or pulmonary embolism in contrast to those without IBD. Furthermore, the risk elevates significantly during a disease flare [7,14,15,27]. This risk appears unique to those with IBD, as demonstrated by Miehsler et al., who showed that other chronic inflammatory conditions, such as rheumatoid arthritis, do not exhibit an elevated risk of thromboembolism [24]. While the overall risk of VTE generally rises with age, the most significant relative risk of VTE in individuals with IBD occurs among patients under the age of 40 [7,9,12,24]. Moreover, the relative risk of VTE during pregnancy is notably higher among those with IBD compared to those without [28]. Additionally, mortality rates linked to VTE are higher in patients with IBD compared to those without the condition [7].

## 3. Pathophysiology

The exact mechanism underlying the heightened VTE risk in individuals with IBD remains to be fully elucidated. This risk is believed to be multifaceted, influenced by genetics and environment, prothrombotic states, and endothelial dysfunction. The chronic inflammation in IBD leads to increased production of pro-inflammatory cytokines, including tumor necrosis factor-alpha (TNF-α), interleukin-1 (IL-1), and IL-6, which in turn triggers heightened expression of tissue factor and the coagulation cascade. The inflammation also amplifies platelet activation and aggregation, contributing to a hypercoagulable state [3,12,29,30]. During active disease, patients often experience deficiencies in natural anticoagulants like antithrombin III and proteins C and S, further predisposing them to thrombosis [3,31].

### 3.1. Risk Factors

VTE risk factors in patients with IBD include both disease-specific factors as well as lifestyle or environmental factors.

#### 3.1.1. Disease Activity

Several disease-specific factors (Table 1) modify the risk for VTE in IBD patients, the most important of which is disease activity. Acute flares of IBD have been associated with an increased VTE risk, likely due to heightened systemic inflammation. In one large cohort study, the overall VTE risk amongst IBD patients was observed to be about three times higher. The risk was even more pronounced during a flare (hazard ratio 8.4, 95% confidence interval 5.5–12.8) compared to periods of remission (hazard ratio 2.1, 95% confidence interval 1.6–2.9), compared to the risk in controls [7]. Bollen et al. performed a single-center retrospective cohort study of 84 IBD patients who developed arterial and/or venous thromboembolism. In the evaluated cohort, 70/84 (83%) patients had experienced venous thrombosis, while 14/84 (17%) patients had a history of arterial thromboembolism. Overall, 60/84 (71%) patients had active disease at the time of thromboembolism, with a larger proportion of patients with arterial thromboembolism (83%) having active disease at diagnosis, versus 73% of those with venous thromboembolism [32]. Elevated platelet count (thrombocytosis) seen in IBD patients may be a surrogate marker of disease activity and possible underlying iron deficiency anemia. Thrombocytosis may contribute to the pro-inflammatory state and microvascular thrombosis [33,34]. Better disease control and treatment of iron deficiency may lead to a reversal of thrombocytosis in IBD patients. One study on rats highlighted the potential role of antiplatelet drugs in patients with IBD [35]. We do not suggest treating reactive thrombocytosis with antiplatelet drugs given the scarcity of data highlighting its efficacy.

#### 3.1.2. Disease Phenotype

Some studies have suggested a potential difference in VTE risk by the type of inflammatory bowel disease. A meta-analysis by Fumery et al. found an increased risk of VTE in patients with IBD (RR 1.96, 95% CI 1.67–2.3) [36]. The risk did not appear to vary between individuals with Crohn’s disease or ulcerative colitis; however, when the studies focused solely on assessing hospitalized patients, the heightened risk of VTE was notably more substantial in individuals with ulcerative colitis. Within the meta-analysis, one of the studies demonstrated that active fistulizing disease in Crohn’s disease patients independently contributed to an increased risk of VTE (OR 1.39, 95% CI 1.13–1.70) [25]. Additionally, a subgroup analysis of those patients with Crohn’s disease showed those with Crohn’s colitis had almost 40% higher likelihood of VTE compared to Crohn’s limited to the small intestine. Interestingly, in the patients with ulcerative colitis, there did not appear to be a statistically significant difference in the rate of VTE compared to those with left-sided disease or proctitis (25.8 per 1000 vs. 21.2 per 1000, *p* = 0.06) [25].

#### 3.1.3. Hospitalization and Surgery

**Figure 2:**
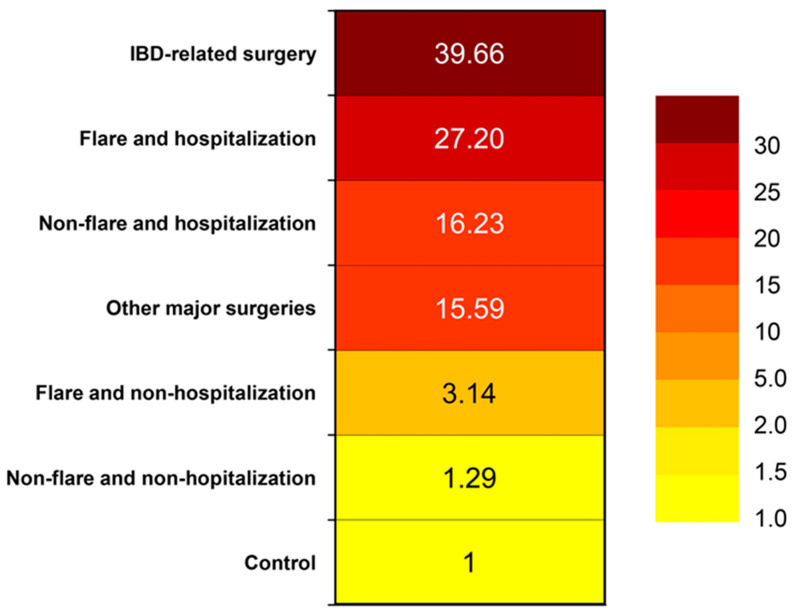
Heat map showing venous thromboembolism risk in a Korean population [26].

Hospitalization, particularly if secondary to an IBD flare or surgery, emerges as a significant factor that notably heightens VTE risk. A retrospective study evaluated hospital discharges and revealed that individuals with ulcerative colitis (OR 1.85, 95% CI 1.70–2.01) or Crohn’s disease (OR 1.48, 95% CI 1.35–1.62) experienced higher VTE rates compared to non-IBD patients who were discharged [25]. Kim et al. conducted a cohort study using Korean National Health Insurance claims data to estimate the risk of VTE over various time frames [15]. The risk of VTE by disease activity and hospitalization was calculated. The risk of VTE during a non-hospitalized flare in IBD patients was higher compared with controls (adjusted HR, 2.86; 95% CI, 1.70–4.80). The risk of VTE increased to a much greater degree during a hospitalized flare (adjusted HR, 19.36; 95% CI, 9.59–39.07) and even during hospitalization without a flare (adjusted HR 12.97; 95% CI, 8.68–19.39). The highest risk of VTE was at the time of IBD-related surgery (adjusted HR, 40.81; 95% CI, 10.16–163.92). The risk of VTE during other major surgeries was increased (adjusted HR, 15.44; 95% CI, 7.65–31.12) [15]. A heat map showing the risk of VTE is shown in Figure 2.

#### 3.1.4. Post-Hospitalization

While VTE prophylaxis is recommended for hospitalized IBD patients, there are no specific guidelines regarding extending this precaution into the early post-discharge period, during which the risk of VTE might remain notably high [37,38]. The reported occurrence of VTE after discharge among IBD patients varies. The decision to provide thromboprophylaxis after hospital discharge is intricate and requires a comprehensive understanding of various factors (Table 2): the absolute risk of VTE, duration of risk, safety, economic considerations, and patient preferences [8]. Ananthakrishnan et al. conducted a retrospective analysis involving multiple medical centers with about 2800 IBD patient hospitalizations, reporting a post-discharge VTE rate of 2% over a six-month period [39]. McCurdy et al., in a single-center Canadian retrospective study with about 2200 IBD hospitalizations, reported a 3% VTE rate within the same time frame [40]. Studies based on population data reported lower rates. For instance, Faye et al., in a U.S. study examining 872,122 IBD hospitalizations, found a VTE readmission rate of 0.13% at 90 days post-discharge [41]. In another Canadian study by McCurdy et al., among 81,900 IBD discharges, a higher cumulative incidence of VTE was found—1.2% among those who underwent surgery and 1.6% among non-surgical patients at 6 months post-discharge [42]. In the context of IBD patients who underwent abdominopelvic bowel surgery, Benlice et al. observed a 1% rate of VTE within 30 days post-discharge among 24,182 hospitalized IBD patients [43]. The patients were assessed utilizing The American College of Surgeons National Surgical Quality Improvement Program Participant (NSQIP) User File [43]. These data suggest that the incidence of VTE after discharge in IBD patients is typically between 1% and 3%. Discrepancies in study outcomes likely stem from differences in patient demographics, follow-up durations, and methods used for event identification [8].

Debates regarding the duration of post-discharge VTE risk persist due to limited research with varying follow-ups. Per the study referenced earlier by Faye et al., it was observed that 91% of VTE readmissions took place within two months post-discharge, with the highest risk within the initial ten days [41]. Studies focusing on post-surgery patients indicated briefer durations of VTE risk. One study found that about 60% of VTE events occurred within two weeks post-discharge, while another study noted that about 65% of VTE instances happened within one month [43,44]. There is a possibility of extended VTE risk in non-surgical IBD patients, potentially associated with persistent inflammation and the use of immunosuppressive therapies [8].

#### 3.1.5. Medications

Corticosteroids have a laundry list of side effects, one of which appears to be an increased risk of VTE. Sarlos et al. performed a meta-analysis that showed corticosteroid use was associated with a higher risk of VTE in IBD patients [45]. One retrospective study indicated that such risk may be dose-dependent, where high-dose corticosteroids showed an association with OR 3.31 (95% CI 2.50–4.37) as opposed to lower doses [10]. There have been suggestions that corticosteroid use could serve as an indirect indicator of disease activity. Therefore, the heightened VTE risk associated with corticosteroids may simply reflect the increased risk accompanying disease flares. However, studies have shown that corticosteroids increase VTE risk in the general population [46], where the risk is thought to be attributable to excess cortisol, as patients with Cushing’s syndrome have an increased risk of VTE due to elevated production of coagulation factors and impaired fibrinolysis [47].

Conversely, anti-TNF agents have been linked to a reduced VTE risk among IBD patients. Although this decrease is likely attributed to decreased disease activity, it is essential to note that TNF has also been associated with thrombus formation and the activation of the coagulation process. Consequently, blocking TNF may serve as a protective factor against VTE [11]. In a retrospective cohort study conducted by deFonseka et al., systemic corticosteroids were associated with a four-fold increased risk of VTE. In contrast, anti-TNF agents exhibited a decreased risk (OR 0.2, 95% CI 0.04–0.99) [48]. Moreover, another study demonstrated a similar finding, noting that patients on biologics had nearly a five-fold lower likelihood of developing VTE compared to individuals receiving corticosteroids [10].

A recent medication class-small molecule inhibitors-has gained traction in recent years. Within this class, JAK inhibitors, particularly tofacitinib, have been linked to an increased VTE risk dependent on dosage [11]. Olivera et al. conducted a meta-analysis that comprised ten controlled studies involving 5143 patients with immune-mediated conditions, including IBD, who were exposed to JAK inhibitors. No significant difference in the risk of VTE was observed with the use of JAK inhibitors [49]. A recent analysis conducted after the fact involving ulcerative colitis patients undergoing treatment with tofacitinib revealed that out of the 1157 patients in the cohort, five patients from the tofacitinib treatment arm developed VTE (one DVT, four PE) as compared to two patients in the placebo arm [50]. It is important to highlight that all patients who experienced VTE did so during the open-label extension study, not during the induction or maintenance studies. These occurrences were observed among those using the higher 10 mg twice daily dosing. Additionally, they had at least one VTE risk factor, such as obesity, hormone replacement therapy, or a prior history of VTE [50]. More data is required to understand these findings further.

#### 3.1.6. Other Risk Factors

Increasing age, obesity, and pregnancy are risk factors associated with VTE risk in the general population and those with inflammatory bowel disease [9,26]. Other risk factors for VTE that are more frequent in IBD patients compared with the general population include indwelling catheters, dehydration, and hyperhomocysteinemia (vitamin deficiency) [12].

While initially thought to be related to hereditary factors, individuals with IBD do not exhibit an increased likelihood of inherited thrombophilia. Consequently, testing for hereditary or acquired hypercoagulable conditions is not advised [37,51]. For instance, the prevalence of factor V Leiden, G0210A mutation in the prothrombin gene, homozygous C677T mutation in the MTHFR gene, or the presence of antiphospholipid antibodies remains comparable among individuals with and without IBD. This similarity also holds when comparing patients with IBD, regardless of VTE occurrence [11,51,52,53].

#### 3.1.7. Mortality amongst IBD Patients with VTE

A few studies have explored the impact of VTE on individuals with IBD [9]. Solem et al. observed a mortality rate of 22% among close to a hundred consecutive IBD patients with VTE, monitored for a median duration of nearly two years [54]. This rate aligned with the 18% mortality rate previously reported within their institution among IBD patients with extremity DVT or pulmonary embolism [55]. Nguyen et al. noted a 2.5-fold (95% CI 1.83–3.43) increased odds of mortality for VTE-related hospitalizations in IBD patients compared to non-VTE-related cases. For non-IBD patients, the presence of VTE was associated with relative odds of in-hospital death of 1.41 (95% CI 1.25–1.58). Adjusting for confounding variables, the excess mortality (odds ratio for death) linked to VTE was two times higher for IBD patients compared to non-IBD individuals (95% CI 1.6–2.9 times, *p* < 0.0001) [25]. Directly comparing these mortality rates with the general population is challenging due to IBD patients’ younger age and lower comorbidity index. The heightened mortality observed among IBD patients experiencing VTE likely involves multiple factors, potentially connected to disease complications and increased susceptibility to infections due to various immunosuppressive agents [9].

#### 3.1.8. VTE and Health Resource Utilization

In the previously mentioned study by Nguyen et al. [25], it was observed that patients with IBD who encountered a VTE had notably extended hospital stays nearly twice as long compared to those without VTE (11.7 days compared to 6.1 days, *p* < 0.0001). The presence of VTE led to a 48% longer hospital stay (95% CI 41–56% increase) after adjusting for demographic, clinical, and hospital-related factors. In addition, undergoing surgery independently correlated with a 110% increase in the duration of hospital stay (95% CI 105–115%). This prolonged hospitalization had a financial impact as well. Total hospital charges were notably higher for IBD patients admitted with a concurrent VTE compared to those without VTE (approximately USD 47,500 compared to USD 21,500; *p* < 0.0001). Even after adjusting for multiple factors, the presence of VTE led to an average 59% increase in total hospital charges (95% CI 51–69%). Specifically, having bowel resection surgery alone increased the average total charges by about 190% (95% CI 181–204%), while being in an urban area, as opposed to a rural setting, increased charges by 30% (95% CI 16–46%) [25].

## 4. Prevention

Guidelines by several societies recommend VTE prophylaxis amongst IBD patients, sometimes varying in recommendation by disease type or severity. For instance, while The American College of Gastroenterology advises VTE prophylaxis using heparin for patients hospitalized with acute severe colitis, an international consensus guideline suggests administering thromboprophylaxis to hospitalized IBD patients, regardless of the reason for hospitalization [11,56]. Nearly all medical societies recommend pharmacological VTE prophylaxis over mechanical prophylaxis, as it is more efficacious [11,12]. The preferred agents usually include low molecular weight heparin (LMWH) and fondaparinux, although unfractionated heparin (UFH) is also recommended. LMWH and fondaparinux are favored due to their lower rates of pulmonary embolism or symptomatic deep vein thrombosis and reduced adverse effects such as significant bleeding or heparin-induced thrombocytopenia compared to UFH [57].

Amid substantial evidence pointing to a notably heightened risk of VTE among IBD patients and societal recommendations for pharmacologic prophylaxis, the actual administration rates of VTE prophylaxis during IBD-related hospitalizations are low. A study from a single center examining all IBD-related VTE cases discovered that merely half of the patients received VTE prophylaxis [58]. Similarly, a study focused on surgical inpatients who experienced IBD-related VTE events revealed that 44% did not receive prophylaxis. Reasons for not administering pharmacologic prophylaxis commonly included gastrointestinal bleeding and ambulatory status [59]. To explore the factors linked to the absence of VTE prophylaxis among hospitalized IBD patients, Faye et al. conducted a retrospective analysis involving nearly 500 IBD patients [60]. The study revealed that IBD patients, particularly those with hematochezia, were less likely to receive VTE prophylaxis (57% with hematochezia compared to 86% without hematochezia). This finding is particularly problematic since 95% of patients with hematochezia were amidst a disease flare, during which the VTE risk increased by more than sixfold [7]. However, neither hematochezia nor VTE prophylaxis showed associations with increased rates of blood transfusions or clinically significant declines in hemoglobin levels during hospitalization [3,60]. The research indicates the safety of thromboprophylaxis in patients with IBD, even when hematochezia is present. Another crucial factor independently associated with decreased VTE prophylaxis was the admission to a medical service instead of a surgical service, leading to a notably lower likelihood of patients admitted to medical service receiving VTE prophylaxis [60]. Previous studies have documented comparable findings, possibly stemming from differences in the protocols employed across these services [61,62]. A meta-analysis examining the safety of heparin in active ulcerative colitis did not find an increased risk of adverse events [63]. All healthcare providers must be informed about the amplified VTE risk in IBD and the safety of pharmacologic prophylaxis in such situations.

In terms of post-discharge prophylaxis, McCurdy et al. proposed a risk assessment tool for IBD patients, incorporating factors such as age (>45 years of age) and length of admission (>7 d) [40]. This scoring system effectively distinguished a specific subgroup of IBD patients who may benefit from post-discharge VTE prophylaxis. This approach would selectively offer post-discharge VTE prophylaxis to those at the highest risk, and their findings indicated that it could help avoid post-discharge VTE prophylaxis in 92% of hospitalized IBD patients. In a different study that investigated factors associated with post-discharge VTE among IBD patients, Faye et al. identified variables like advanced age, discharge to a skilled nursing facility, and a prior history of *C. difficile* upon initial admission as factors that heightened this risk [60]. Additionally, they found that more than 90% of VTE-related readmissions occurred within 60 days after discharge, with the majority occurring within the initial 20 days. In order to further assess the benefit of post-discharge prophylaxis, studies examining the cost-effectiveness of post-discharge prophylaxis among high-risk IBD patients are needed. In addition to cost, continued prophylaxis’s benefits must be weighed against the risk of bleeding and polypharmacy [3].

For individuals needing significant surgical interventions, the 2018 Enhanced Recovery After Surgery (ERAS) guidelines for perioperative care during elective colorectal surgery suggest employing a series of measures [64]. These include utilizing mechanical thromboprophylaxis with intermittent pneumatic compression or appropriately sized compression stockings until the patient is discharged and administering LMWH pharmacological prophylaxis once a day for 28 days after the surgery.

## 5. Treatment

Limited research covers the treatment of VTE in IBD patients. Treatment for VTE in IBD patients is similar to those for individuals without IBD [65,66]. Historically, it has been recommended that unless there is significant bleeding or a requirement for thrombolysis, the preferred treatment is LMWH, often transitioning to warfarin.

The ideal duration for anticoagulant therapy remains uncertain, demanding a careful balance between the risk of recurrent VTE in IBD patients and the bleeding risk associated with anticoagulation [12]. Novacek et al. observed that among IBD patients experiencing their first unprovoked VTE, there was a 33% chance of a subsequent episode within half a decade. This represented a 2.5 times higher risk of recurrence compared to non-IBD patients after an initial unprovoked VTE [26]. Regarding the duration of therapy, Nguyen et al. conducted a decision analysis study that compared time-limited (6 months) and longer-duration anticoagulation for managing VTE in IBD. The study concluded that in IBD patients with a history of unprovoked VTE, the benefits of prolonged anticoagulation in preventing recurrent VTE outweighed the associated risks of bleeding, particularly for patients who experienced an unprovoked VTE in the absence of a flare or other temporary risk factors [67]. There have been documented instances of successful catheter-directed thrombolysis in patients with IBD [68]. Inferior vena cava filters are indicated for cases involving thrombi in deep leg veins that may embolize, recurrent PE despite anticoagulation, or a high risk of bleeding [69].

In recent times, significant shifts have occurred in anticoagulation treatment choices, primarily attributed to the advent of direct oral anticoagulants (DOACs) [70]. These medications, which include direct factor Xa inhibitors (such as rivaroxaban, apixaban, and edoxaban) and a thrombin inhibitor (dabigatran), bring several advantages to the table, such as obviating the need for INR monitoring or heparin bridging. DOACs might facilitate earlier home-based treatment for stable VTE patients compared to warfarin. They might also surpass vitamin K antagonists in treating VTE and preventing post-thrombotic syndrome [71]. Some reports even indicate that using DOACs to treat acute symptomatic VTE leads to notably lower risks of overt bleeding compared to vitamin K antagonists [72]. However, it is essential to note that these medications lack readily available reversal agents in the case of overdose. Further information is required to determine the use of these medications in relation to IBD. DOACs might have a particularly valuable role in managing outpatients with IBD. Controlled trials are necessary to confirm their potential benefits over warfarin in IBD patients experiencing VTE [70].

## 6. Conclusions

Patients diagnosed with IBD face a notably higher risk of VTE, leading to significant health challenges and even fatalities [3,7]. Multiple factors contribute to this escalated risk, including inflammation and its effects on the coagulation cascade [30]. Various clinical factors, such as age, disease phenotype, severity, hospitalization, pregnancy, surgical interventions, and specific medications like corticosteroids, all elevate the chances of experiencing VTE. While the societal guidelines offer varying recommendations for non-IBD-related admissions, they unanimously suggest VTE prophylaxis, ideally using pharmacological methods, for patients admitted due to an IBD flare without hemodynamically unstable bleeding. However, adherence to these recommendations, whether within community healthcare settings or academic medical centers, tends to be low, often due to concerns regarding the safety of chemical prophylaxis in situations involving hemodynamically stable gastrointestinal bleeding, despite the evidence to the contrary. Diverse therapeutic options, including small molecule drugs such as upadacitinib, continue to emerge. Further long-term studies are needed to assess their safety, particularly their impact on VTE risk. We hope that the data above leads the reader to protect patients from a possibly deadly complication.

## Figures and Tables

**Table 1:** Factors associated with increased risk of VTE.

Venous Thromboembolism Risk Factors	
Disease-Specific Factors	Patient-Specific Factors
Disease activity (flares)	Advanced age
Disease phenotype	Obesity
Hospitalization	Pregnancy
Surgery	Indwelling catheters
Corticosteroid use	Hyperhomocysteinemia

**Table 2:** Factors influencing post-discharge thromboprophylaxis in IBD patients.

Factors Influencing Post-Discharge Thromboprophylaxis
Absolute risk of VTE
Duration of risk
Safety
Economic considerations
Patient preferences
